# Toll-like receptor 4-driven translational reprogramming in macrophages is unaffected by *Salmonella* SPI-1 activity

**DOI:** 10.1016/j.isci.2026.117901

**Published:** 2026-10-26

**Authors:** George Wood, Rebecca Johnson, Matthew P. Brember, Filip Lastovka, Panagiotis Tourlomousis, Clare E. Bryant, Betty Y.-W. Chung

**Affiliations:** 1 Department of Pathology, University of Cambridge, Cambridge, UK; 2 Department of Medicine and Department of Veterinary Medicine, University of Cambridge, Cambridge, UK

**Keywords:** translation control, host-pathogen interaction, TLR4, SPI-1 injectisome/ribosome profiling

## Abstract

Changes in gene expression during bacterial infection are the combined result of altered transcription and translation, with the latter comparatively understudied. Gram-negative bacteria rapidly trigger cytokine gene transcription in macrophages through the activation of pathogen-associated molecular pattern receptors, for example the detection of *Salmonella* lipopolysaccharide (LPS) from the bacterial cell envelope by Toll-like receptor 4 (TLR4). Here, through time-resolved parallel translatomic and transcriptomic profiling, we now show temporal TLR4-specific translational upregulation of cell signaling proteins in macrophages induced by *Salmonella*. While transcriptional upregulation of these genes is dampened through the activity of the *Salmonella* SPI-1 type 3 secretion system, a robust translational response remains. These data reveal an important host-pathogen translational regulatory network that modifies the innate immune response of macrophages to infection.

## Highlights

• *Salmonella*-macrophage interaction triggers rapid translational reprogramming
• SPI-1 injectisome suppresses TLR4-dependent transcriptional responses
• TLR4-driven translational activation remains intact despite SPI-1 interference

## Graphical abstract

**Figure:**
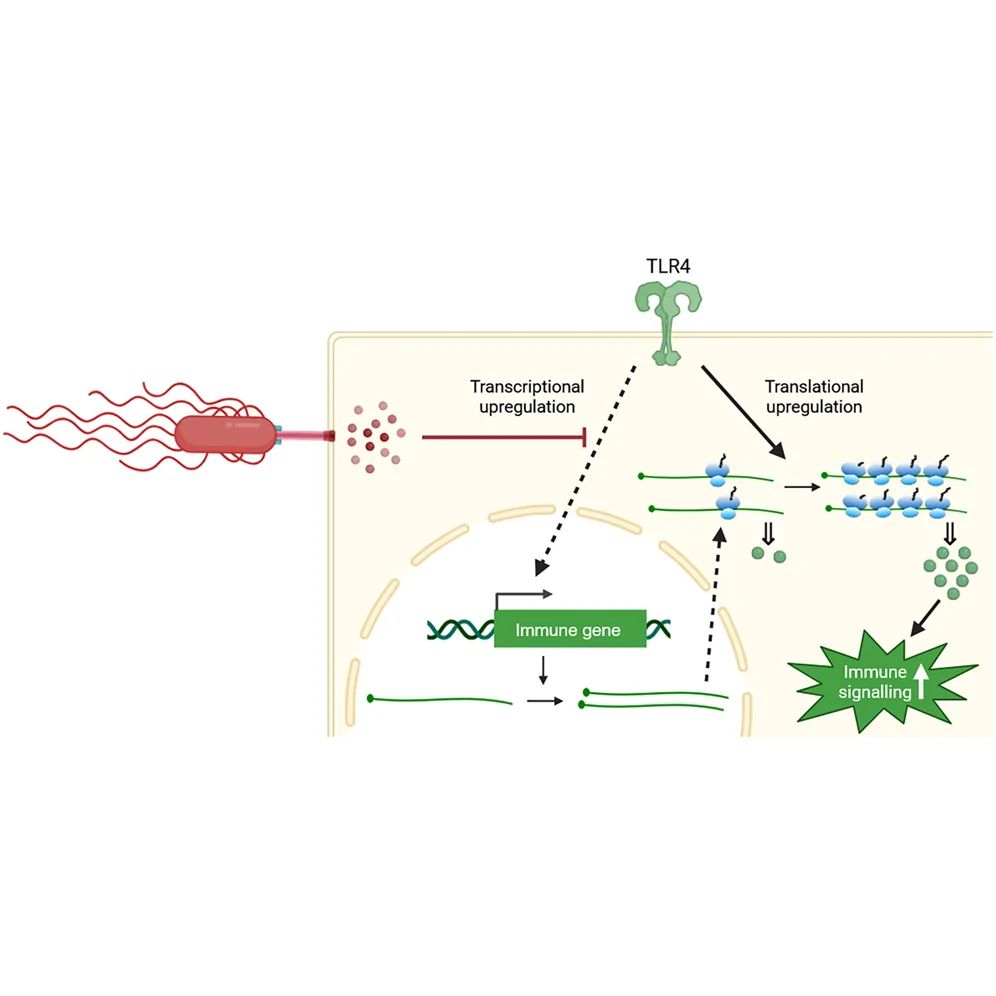

## Teaser

biological sciences; microbiology; bacteriology

## Introduction

Rapid activation of the innate immune response is among the first lines of defense against invading pathogens and plays a crucial role in controlling and eliminating infection. Pathogen-associated molecular patterns (PAMPs) are detected by germ-line-encoded host receptors, triggering an innate immune response. Toll-like receptors (TLRs) detect a variety of PAMPs. This includes bacterial lipopolysaccharide (LPS), lipoproteins and flagellin, which are detected by TLR4, TLR2 (in combination with TLR1 or TLR6), and TLR5, respectively.^1^ Their activation in innate immune cells induces anti-microbial defenses and triggers cytokine and chemokine production for recruitment and modulation of further immune cells to aid in fighting the infection.^2^ As such, pathogen detection triggers dramatic alteration of host cell protein synthesis. The transcriptional response to bacterial pathogens is well studied, but our understanding of gene expression is incomplete without examining translational regulation also. While purified bacterial PAMPs have been shown to modulate translation of specific genes independently of transcription,^3^^,^^4^ there are few studies that dissect the TLR4-specific transcriptional response from the translational response to bacterial infection.

*Salmonella* is a Gram-negative bacterial pathogen that can infect many different host cells and cause a range of diseases, from typhoid fever to gastroenteritis.^5^ On infection, *Salmonella* pivots to an intracellular life cycle, supported by two type 3 secretion systems encoded on *Salmonella* pathogenicity islands (SPIs) 1 and 2. SPI-1 is important for bacterial invasion of host epithelia, but macrophages can additionally take up bacteria by phagocytosis, whereas SPI-2 is expressed once the bacterium is intracellular to support *Salmonella* survival and replication.^6^ Both secretion systems also deliver effectors that modulate host cell immune signaling pathways.^6^^,^^7^^,^^8^^,^^9^^,^^10^^,^^11^ TLR4 signaling is of key importance in *Salmonella* infection, where it plays roles in both infection control and bacterial virulence.^12^^,^^13^^,^^14^

The main PAMP recognized by TLR4 is LPS, which is found on the outer membrane of Gram-negative bacteria, such as *Salmonella*. LPS binds to TLR4 and its co-receptor MD2^15^ with intracellular signaling orchestrated by homotypic interactions with the TLR4 Toll/interleukin-1 receptor (TIR)-domains and the TIR-containing adaptor proteins TIRAP and TRAM. These proteins form scaffolds for the binding of the TIR-containing MyD88 and TRIF, respectively.^16^ TLR4 at the cell surface acts through the TIRAP-MyD88 pathway triggering many downstream signaling pathways including the activation of mitogen-activated protein kinase (MAPK) and NF-κB. The activation of MyD88-dependent signaling is shared with the other TLRs except for TLR3.^1^^,^^16^^,^^17^ LPS stimulation also triggers TLR4 internalization from the cell surface into endosomes.^18^^,^^19^^,^^20^ Endosomal TLR4 signals through the TRAM/TRIF pathway leading to activation of alternative signaling pathways, including through IRF3.^18^^,^^20^ The TRIF-dependent signaling pathway is shared with TLR3.^1^^,^^17^

TLR4 stimulation leads to significant transcriptional and translational reprogramming as part of the inflammatory response.^3^^,^^4^ Activation of inflammatory transcription factors, including NF-κB and IRF3, leads to the transcription of immune response genes, including cytokines that signal to other immune cells. The TLR4-dependent translational response is comparatively less studied. Through the use of purified LPS to activate TLR4, it is known that translational upregulation of select mRNAs, including those encoding cytokines and negative feedback modulators of the inflammatory response, downstream of TLR4 activation is achieved by modulating the activity of RNA-binding proteins that recognize *cis*-acting elements within these transcripts.^4^^,^^21^^,^^22^ In addition, changes in the translation of factors involved in mitochondrial oxidative phosphorylation have also been reported following TLR4 stimulation, leading to LPS-induced decreases in macrophage metabolism.^3^

Rapid translational changes upon *Salmonella*-macrophage infection that are dependent on the SPI-1 injectisome have been previously identified.^23^ This includes SPI-1 injectisome-dependent translational enhancement of genes whose expression is classically considered to be TLR4-dependent, such as pro-IL-1β, which showed clear SPI-1 injectisome-dependent expression within 60 min.^20^^,^^24^^,^^25^^,^^26^ *Salmonella* infection-dependent differences in translation could be detected as early as 5 min post-infection^23^ and morphological changes in macrophages following TLR4 stimulation have also been observed in this time frame.^27^ Therefore, the first hour of macrophage infection was closely inspected to identify TLR4-dependent changes in gene expression utilizing knockouts of both host TLR4 and the bacterial SPI-1 machinery. Gene expression was assayed at the levels of RNA abundance, using RNA sequencing (RNA-seq) and protein synthesis, using ribosome profiling (Ribo-seq). Ribo-seq is a highly sensitive method that reveals the global translatome at the time of harvest.^23^^,^^28^ The technique determines the position of ribosomes by exploiting the protection from nuclease digestion of a discrete fragment of mRNA (∼30 nucleotides) conferred by a translating ribosome. Deep sequencing of these ribosome-protected fragments (RPFs) generates a high-resolution view of the location and abundance of translating ribosomes on different mRNA species, reflecting the amount of synthesis of specific proteins. This work highlights the ongoing host-pathogen battle where *Salmonella* suppresses host inflammatory protein production through transcriptional suppression, but this is circumvented by a rapid host translational response.

## Results

### TLR4 stimulation triggers rapid changes in macrophage morphology and cytokine production

*Salmonella* infection of macrophages is known to stimulate host actin polymerization resulting in ruffling of the cell membrane.^29^^,^^30^ Both wild-type (WT) *Salmonella* and a strain unable to produce a functional SPI-1 injectisome, Δ*prgJ*^31^^,^^32^^,^^33^^,^^34^—hereafter the injectisome mutant— induce ruffling in WT immortalized bone marrow-derived macrophages (iBMDMs) to similar levels within 15 min (Figures 1A and 1C). Macrophages lacking TLR4, on the other hand, exhibit ruffling when infected by WT *Salmonella* but not with *Salmonella* deficient in SPI-1 secretion (Δ*prgJ*). Notably, TLR4-deficient macrophages display an even higher proportion of ruffled cells than WT macrophages following WT *Salmonella* infection. This increased ruffling likely reflects the absence of resolution mechanisms that normally act downstream of TLR4.^35^ Stimulation with Pam3CSK4 (TLR2/1 agonist), flagellin (TLR5 ligand), or inert beads does not induce ruffling in either WT or TLR4-deficient macrophages, whereas LPS induces ruffling in a TLR4-dependent manner^27^ (Figure 1B). This suggests that two independent mechanisms drive the rapid changes in macrophage morphology during *Salmonella* infection: an LPS-TLR4-dependent and an SPI-1 injectisome-dependent mechanism.

**Figure 1:**
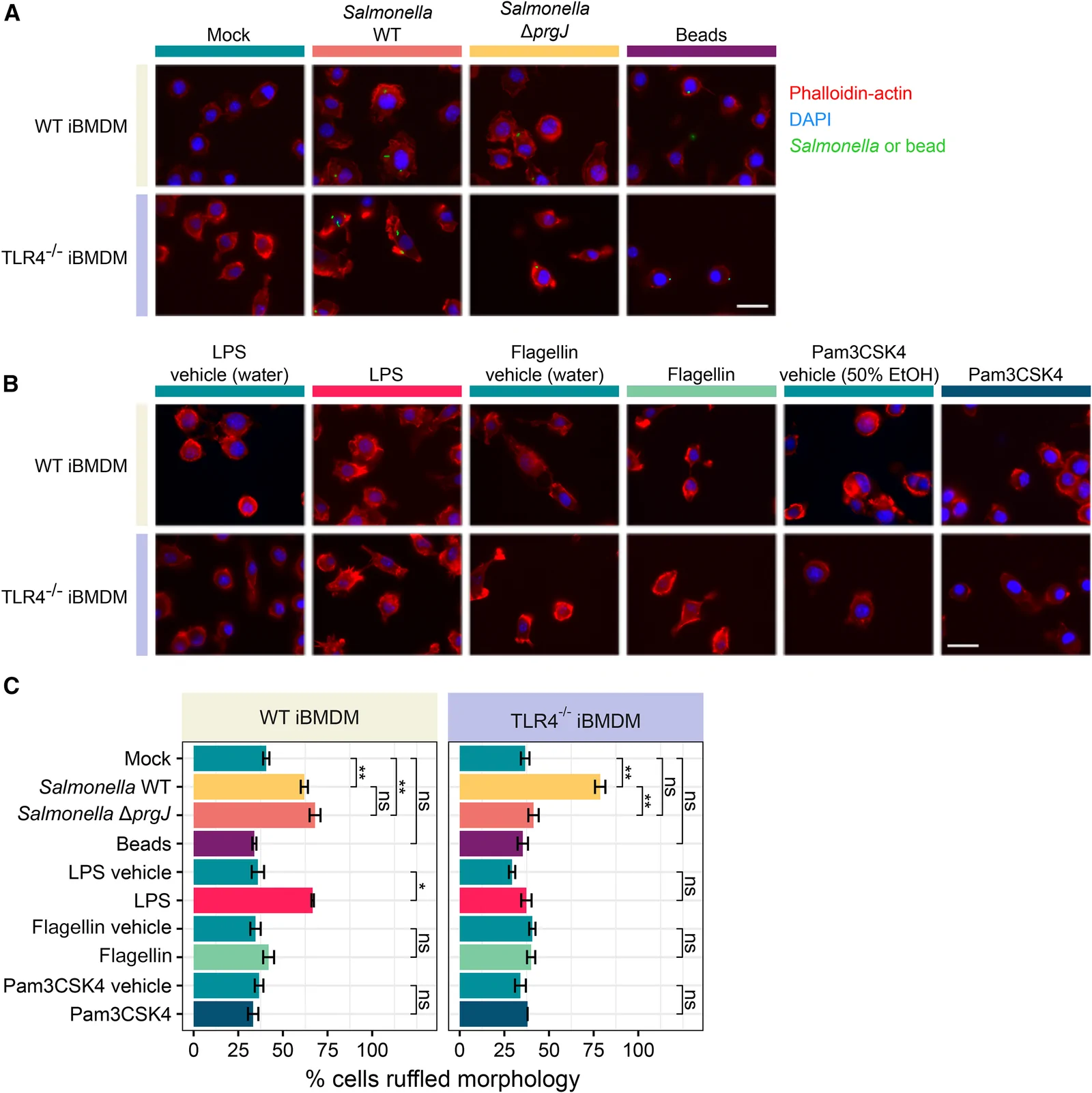
Infected macrophages undergo rapid TLR4-induced morphological changes during *Salmonella* infection (A and B) Representative images of stimulated macrophages, 15 min post stimulation. Phalloidin-actin is shown in red; DAPI is shown in blue; and bacteria or beads are shown in green. Scale bars, 20 μm. (A) Cells stimulated with either WT or SPI-1 injectisome-deficient (Δ*prgJ*) *Salmonella* at MOI 10, or with inert fluorescent beads at 100 beads per cell, or mock-stimulated cells. (B) Cells stimulated with TLR agonists: 500 ng/mL LPS, 500 ng/mL Pam3CSK4, 40 ng/mL flagellin or the corresponding vehicle: water, 50% ethanol and water, respectively. (C) Bar graph showing percentage of macrophages with ruffled morphology after 15 min of stimulation. *p* values determined by Benjamini–Hochberg adjusted one-sided Student’s *t* test, ns indicates not significant, ∗*p* value <0.05 and ∗∗*p* value <0.01. Data are represented as mean ± SEM, *n* = 3.

Host cell death is characteristic of *Salmonella*-macrophage infection across mice and humans despite macrophages being a major host cell in systemic infection.^36^^,^^37^^,^^38^ Rapid macrophage death is attributed to the activation of the host inflammasome by effectors secreted through the SPI-1 injectisome, triggering pyroptosis^37^^,^^38^^,^^39^ and is independent of TLR4 activation (Figure S1A). As most infected macrophages rapidly die (>50% within the first 60 min and >80% by 4 h), they will have a limited window in which they can produce proteins associated with innate immunity to coordinate the response to infection.

Given this limited time frame, we hypothesize that targeted translational upregulation plays a critical role in rapidly producing proteins required for the innate immune response to *Salmonella* infection. Translational regulation can act on pre-existing mRNAs or enhance synthesis from newly transcribed, low-abundance mRNAs, enabling a faster response than transcriptional regulation alone.

It is clear that TLR4 stimulation rapidly alters the host cell (Figure 1) and drives much of the previously characterized innate response to Gram-negative bacteria, such as stimulating the production of cytokines, including IL-1β (Figure S1B). IL-1β secretion is typically thought to require two signals: a priming signal to induce expression of its precursor and a secondary signal to trigger precursor maturation, as occurs following inflammasome activation.^40^ In *Salmonella* infection, the priming step is typically thought of as due to TLR4-mediated transcriptional induction of *Il1b*, though we recently also showed the SPI-1 injectisome played a role in further increasing expression at the translational level.^23^ TLR4 stimulation by LPS has also been shown to increase the translational efficiency (TE) of transcripts encoding cytokines, such as *Tnf*, and other mRNAs containing AU-rich elements in their 3′ UTRs.^4^ However, the extent of the rapid TLR4-dependent translational response during infection, and whether this layer of regulation is actively perturbed by bacteria, remains limited. Therefore, it is important to uncouple TLR4-dependent and independent gene expression responses within the context of *Salmonella* infection. In order to dissect the TLR4-dependent transcriptional response from the translational response, time-resolved parallel RNA-seq and Ribo-seq was conducted on infected WT or TLR4^−/−^ primary BMDMs, hereafter referred to as BMDM^WT^ and BMDM^TLR4^, respectively, with either WT or injectisome-mutant *Salmonella* (Figure 2A).

**Figure 2:**
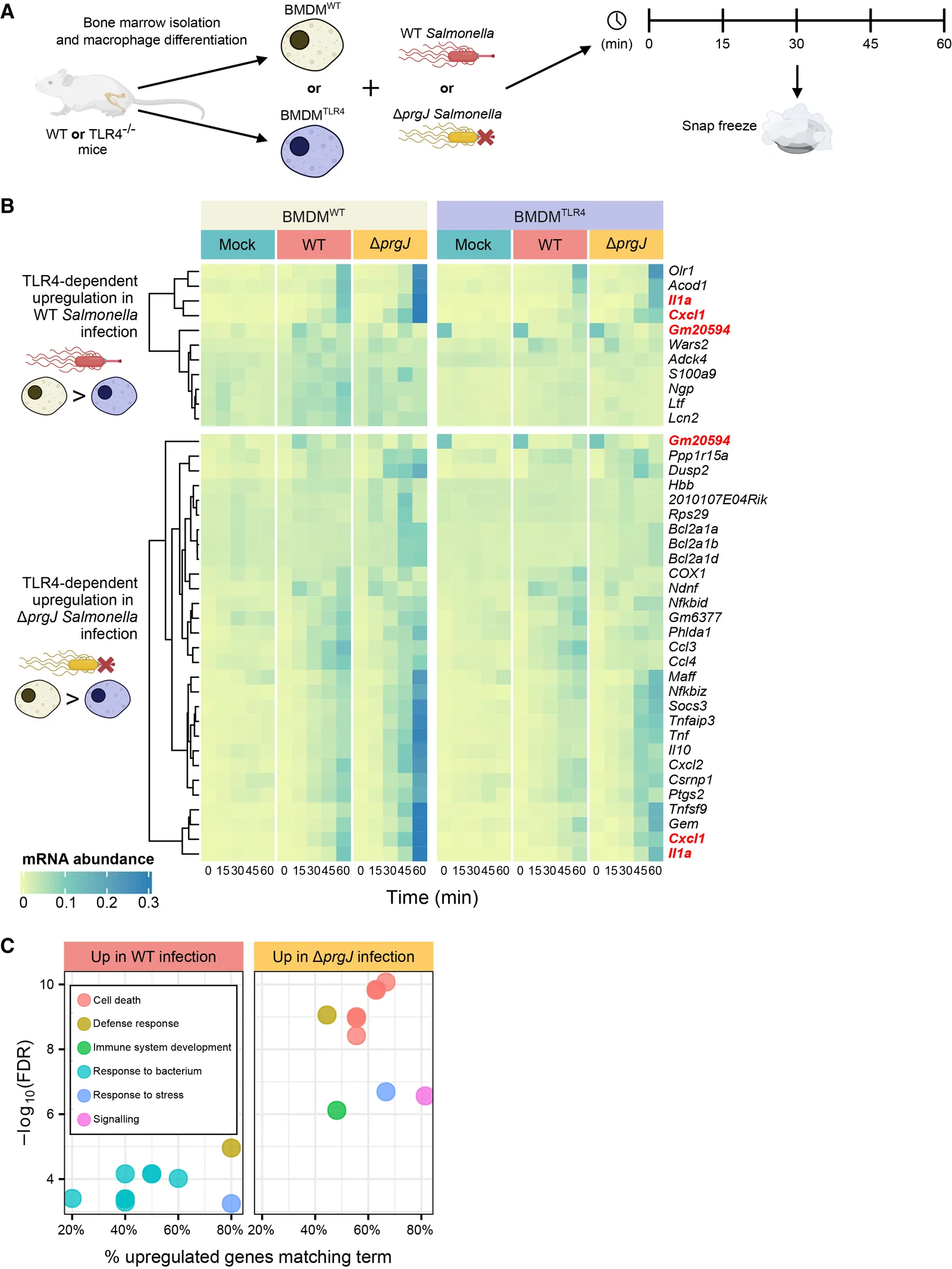
Transcripts upregulated after *Salmonella* infection of BMDM^WT^ over BMDM^TLR4^ are enriched for immune processes Induction is greater when infected with *Salmonella* deficient in SPI-1 effector secretion. Data from two replicates. (A) *Salmonella*-infected BMDM^WT^ or BMDM^TLR4^ were flash frozen every 15 min for the first hour of infection from which parallel RNA- and Ribo-seq libraries were prepared. (B) Heatmaps showing relative RNA abundance of genes with upregulated RNA abundance (log_2_FC > 1) in (top) WT and (bottom) Δ*prgJ Salmonella* infection of BMDM^WT^ over BMDM^TLR4^ that are also upregulated in infection over mock infection of BMDM^WT^ at two or more time points. Values are relative to total normalized RNA-seq counts of that gene across all conditions and time points, that is normalized read counts for each gene are scaled by the sum of normalized counts for that gene across all RNA-seq libraries. Heatmaps use the same color scale. Genes upregulated in both infections are highlighted in red. Order of genes determined by hierarchical clustering of their mRNA abundance across conditions and time points (left). (C) Ten most significantly (smallest adjusted *p* value) enriched GO biological process terms of these differentially expressed genes, manually grouped by similarity. See Tables S1 and S2 for the full list of significantly enriched GO terms. Figures produced with R and BioRender.

### Infected macrophages lacking TLR4 show reduced transcriptional upregulation of defense response genes

Previous work has highlighted that the translational response precedes the transcriptional response in *Salmonella*-macrophage infection, with the translational response dominating over the first hour,^23^ following which the majority of the host macrophages die (Figure S1A). Here, upregulation was defined as having log_2_-fold change (log_2_FC) greater than 1. The *Salmonella* injectisome and its effectors are known to influence the host transcriptional response^6^^,^^7^^,^^8^^,^^9^^,^^10^^,^^11^ and so, to identify the SPI-1-independent TLR4-dependent response, the infection was also conducted using the injectisome-mutant *Salmonella*.

While transcriptional responses are expected to be minimal within this time frame,^4^^,^^23^ the few mRNAs whose abundance is rapidly TLR4-dependently upregulated were identified. Initially, genes with increased transcript abundance in WT *Salmonella*-infected BMDM^WT^ over BMDM^TLR4^ at any time point were selected, filtering out infection-independent differences by requiring them to also be upregulated at more than one time point in WT *Salmonella*-infected over mock-infected BMDM^WT^. This identified 11 genes with both TLR4- and *Salmonella* infection-dependent upregulation in the first hour of infection (Figure 2B, top). *Acod1*, *Olr1*, *Il1a*, and *Cxcl1* showed a gradual increase in mRNA abundance over the first hour of both WT and injectisome-mutant infection. In the absence of host TLR4, this increase is substantially reduced but some persists, indicating that transcriptional upregulation is also driven by other pathways activated by *Salmonella*. The other genes identified as TLR4-dependently upregulated by WT *Salmonella* infection exhibit relatively minor changes over the infection time course, suggesting that their elevated abundance primarily reflects baseline TLR4 signaling rather than dynamic upregulation to ongoing *Salmonella* infection.

*Acod1*, *Olr1*, *Il1a*, and *Cxcl1* all showed greater increase when infected by the injectisome-mutant *Salmonella*. Therefore, genes with TLR4-dependent upregulated transcript abundance in injectisome-mutant *Salmonella* infection were also examined. A total of 29 genes were identified to be TLR4-dependently upregulated in infection without an intact injectisome, using the same selection criteria as for WT *Salmonella* infection. Transcription of 13 of these genes (*Maff*, *Nfkbiz*, *Socs3*, *Tnfaip3*, *Tnf*, *Il10*, *Cxcl2*, *Csrnp1*, *Ptgs2*, *Tnfsf9*, *Gem*, *Cxcl1*, and *Il1a*) was greatest in injectisome-mutant *Salmonella* infection (Figure 2B, bottom). While only 8% of identified genes were shared between WT and Δ*prgJ Salmonella* infection, the majority are upregulated in both but with a fold change less than 2 as used to identify them. Gene Ontology (GO) term enrichment analysis of the genes showing TLR4-dependent upregulation in WT or injectisome-mutant *Salmonella* infection reveals that, as expected, they have roles in immune system processes and the defense response (Figure 2C; Tables S1 and S2). Enrichment analysis also highlighted the TLR4-dependent upregulation of genes involved in apoptosis in injectisome-mutant *Salmonella* infection. This is driven by genes encoding BCL2A1 family proteins, which are anti-apoptotic.^41^ Interestingly, the upregulation of these genes was only seen in the injectisome-mutant *Salmonella* infection, where no host cell death is observed in this time frame (Figure S1A).

### TLR4-induced changes in protein synthesis are not solely due to change in transcription

As described previously, LPS stimulation has been shown, using polysome profiling, to alter the translation activity of macrophage-like cell lines such as J774A.1 and RAW264.7.^3^^,^^4^ This was also demonstrated in iBMDMs in this study (Figure S4A). Nevertheless, the stimulation of TLR4 during an infection cannot be fully recapitulated by purified LPS in solution alone. Additionally, detection of other PAMPs in infection may influence TLR4 signaling and bacterial effectors are known to modulate host immune signaling pathways.^6^^,^^7^^,^^8^^,^^9^^,^^10^^,^^11^^,^^42^^,^^43^ Therefore, TLR4-dependent changes in protein synthesis during *Salmonella* infection were assessed through parallel Ribo-seq on the same samples as for RNA-seq. Due to the high resolution of the data that we obtained, we were able to accurately quantify protein synthesis by only considering the in-frame reads that map to the coding sequence of transcripts, reflecting the movement of ribosomes in three-nucleotide increments during the decoding process, prior to downstream analysis. Genes with increased protein synthesis were similarly identified as for RNA-seq.

A total of 44 and 46 genes exhibited TLR4-dependent increases in protein synthesis in WT and injectisome-mutant *Salmonella* infection, respectively (Figures 3A and 3B). There was a greater overlap in genes showing TLR4-dependent increases in protein synthesis between infection with either WT or Δ*prgJ Salmonella* than in the RNA-seq data, with 27% of genes identified as upregulated in both conditions. GO enrichment analysis of genes with increased protein synthesis identified similar roles to those with increased mRNA abundance: the response to bacteria and the defense response. Enriched cell death-associated GO terms were specific to genes upregulated in Δ*prgJ Salmonella* infection (Figure 3C). Significantly more genes were identified as upregulated in protein synthesis than RNA abundance, i.e., the increases in protein synthesis for upregulated genes cannot be explained by increases in RNA abundance alone (Figures 3D, S2, and S3), highlighting their TLR4-dependent regulation at the level of translation.

**Figure 3:**
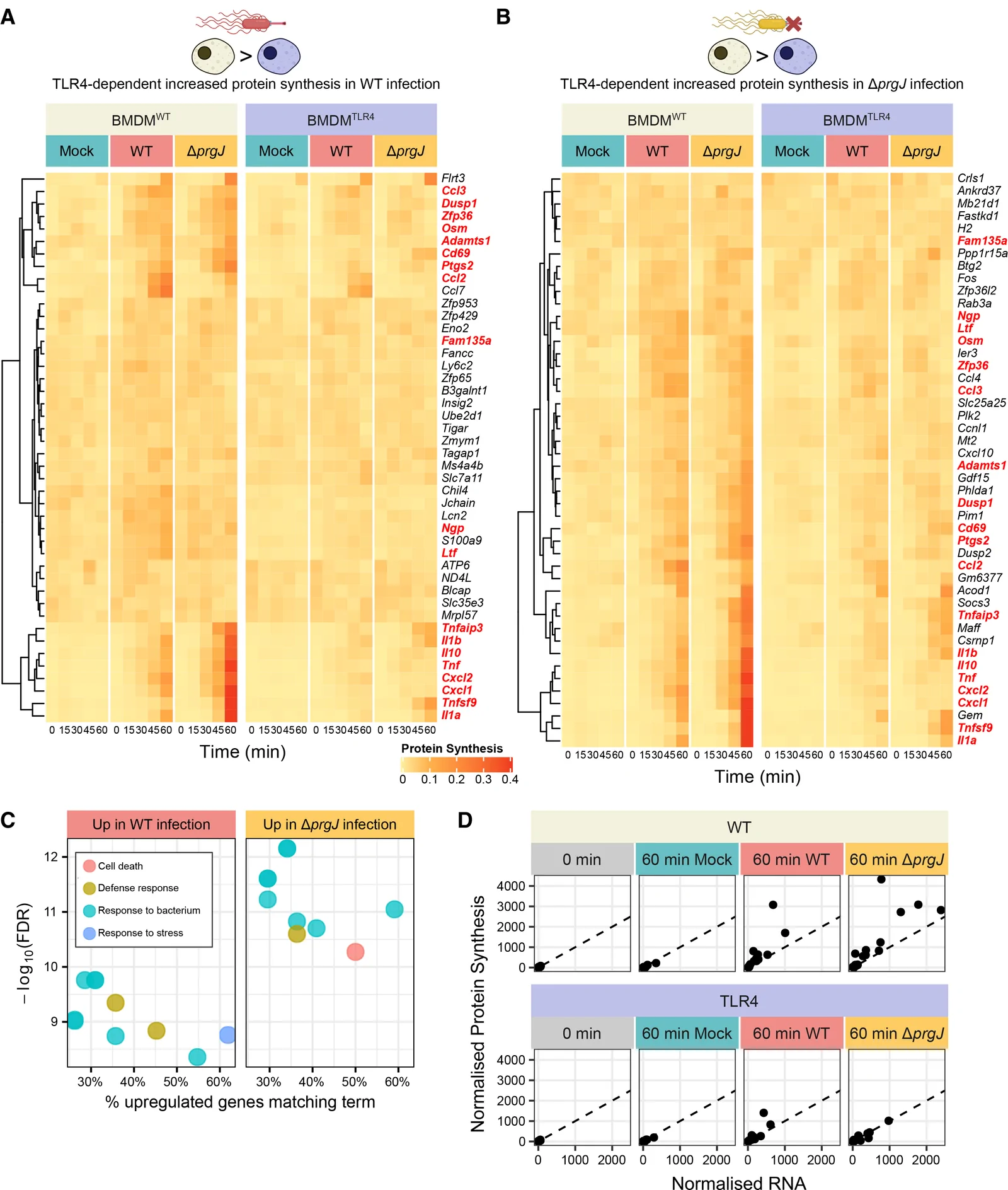
TLR4-dependent changes in protein synthesis are not always dictated by changes in mRNA abundance Data from two replicates. (A and B) Heatmaps showing relative protein synthesis of genes with upregulated protein synthesis (log_2_FC > 1) in (A) WT or (B) Δ*prgJ Salmonella* infection of BMDM^WT^ over BMDM^TLR4^ that are also upregulated in infection over mock infection of BMDM^WT^ at two or more time points. Values are relative to total normalized Ribo-seq counts of that gene across all conditions and time points. Heatmaps use the same color scale. Genes upregulated in both infections are highlighted in red. Order of genes determined by hierarchical clustering of their protein synthesis across conditions and time points (left). (C) Ten most significantly (smallest adjusted *p* value) enriched GO biological process terms of these differentially expressed genes, grouped manually by similarity. See Tables S3 and S4 for the full list of significantly enriched GO terms. (D) Scatterplot comparing normalized RNA abundance and protein synthesis at 0 and 60 min post-infection for genes with TLR4-dependent upregulated protein synthesis in both WT and Δ*prgJ Salmonella* infection (red genes in A and B). Dashed line shows where RNA and protein synthesis are equal.

### TLR4-dependent translational upregulation of cell-signaling genes

The amount of protein produced will depend on both the abundance of the encoding mRNA and the efficiency of its translation, which is independently regulated and occurs within the cytoplasm (Figure 4A). Therefore, TE was determined for all transcripts by comparison of the parallel RNA-seq and Ribo-seq data.^23^^,^^28^ Ribo-seq captures the positions and abundance of translating ribosomes and so, in combination with RNA-seq, can be used to determine the relative number of ribosomes per transcript, a measure of TE. As with the quantification of protein synthesis, the high proportion of highly phased reads in our data (Figure S4B) enables us to only consider in-frame reads, i.e., reads which genuinely reflect ribosome occupation, enabling accurate quantification of TE for all downstream analyses.

**Figure 4:**
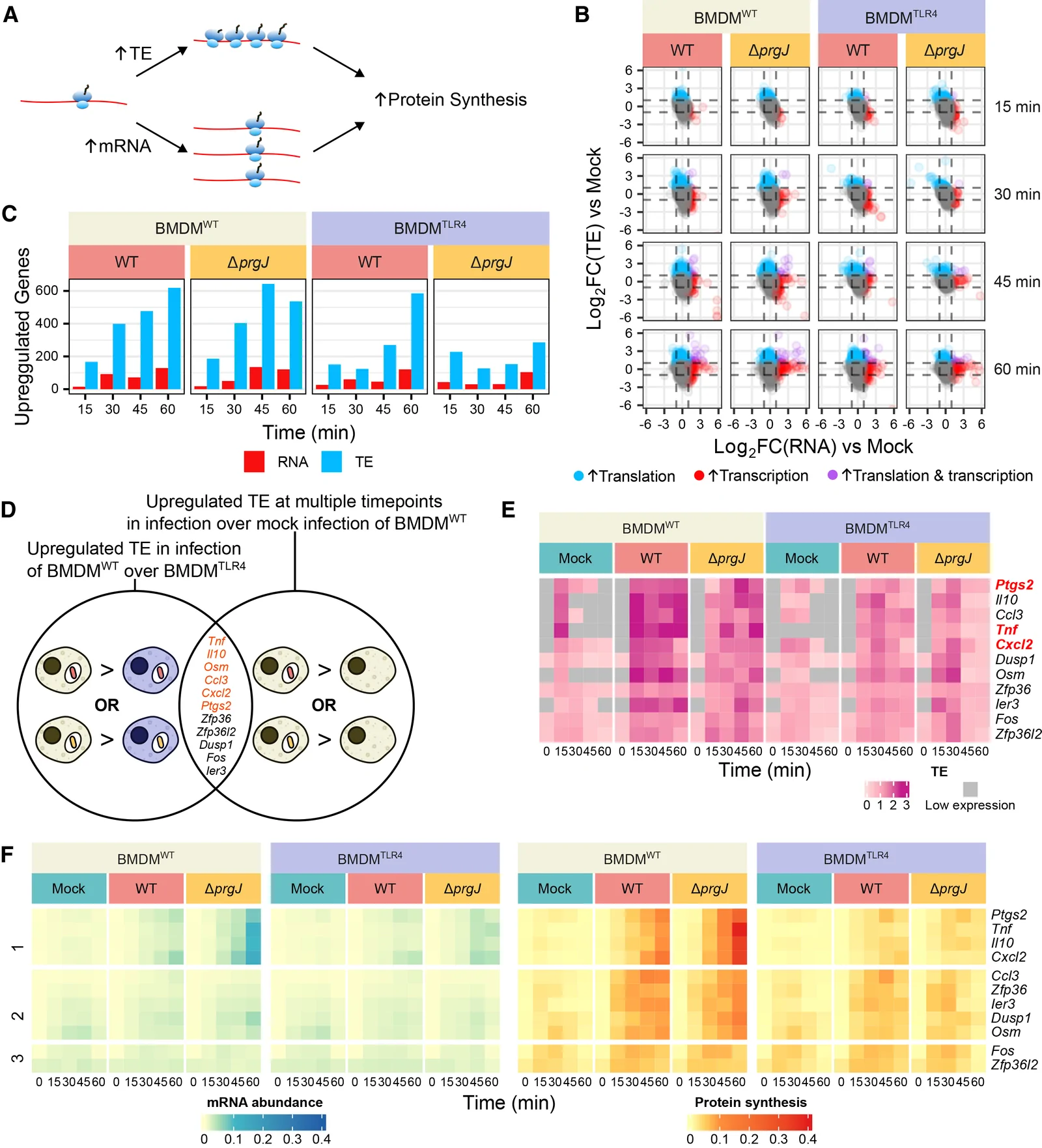
The TLR4-dependent, bacterial-effector-independent translational response Data from two replicates. (A) Schematic showing two routes to increased synthesis of a particular protein: by increasing the number of its mRNAs or by increasing their translational efficiency. (B) Scatterplot comparing log_2_FC in RNA abundance and TE for the indicated condition vs. mock-infected host cells at the same time point. Dashed lines indicate log_2_FC vs. mock of 1 and −1. Upregulated genes are colored accordingly. (C) Bar plot showing number of genes upregulated transcriptionally or translationally. (D) Candidate selection criteria; genes were selected if they were upregulated at any time point in WT or Δ*prgJ Salmonella*-infected BMDM^WT^ over BMDM^TLR4^ and were differentially expressed at 2 or more time points in infection of BMDM^WT^. Genes with direct roles in cell-to-cell signaling are colored orange. (E) Heatmap showing TE of genes selected in (D). An expression cutoff of 10 reads per sample was used for both RNA and protein synthesis, below which TE could not be calculated reliably. Genes in red were upregulated in WT *Salmonella* infection, whereas all genes listed were upregulated in Δ*prgJ Salmonella* infection. (F) Normalized relative RNA- and Ribo-seq counts, used to infer mRNA abundance and protein synthesis levels, respectively, for genes in (D). Values are relative to total normalized RNA- and Ribo-seq counts of that gene. K-means clustering (3 centers) of Ribo-seq counts was used to group transcripts by translational behavior throughout the infections.

Differential analysis of both TE and RNA abundance upon infection demonstrates that variation in gene expression is predominantly driven by changes at the level of translation in the first 60 min of infection, with a greater number of genes upregulated translationally than transcriptionally in all conditions, aligning with our previous report^23^ (Figures 4B and 4C). The combined absence of host TLR4 and bacterial SPI-1 injectisome substantially diminished this translational response, indicating that the two are the major drivers of the early gene expression changes in *Salmonella*-infected macrophages.

Genes with significantly upregulated TE in BMDM^WT^ over BMDM^TLR4^ infected with either WT or injectisome-mutant *Salmonella* were identified and filtered to select those also significantly upregulated in infection vs. mock-infection of BMDM^WT^ at more than one time point (Figure 4D). Eleven genes with TLR4-dependent translational upregulation were identified, namely *Ccl3*, *Cxcl2*, *Dusp1*, *Fos*, *Ier3*, *Il10*, *Osm*, *Ptgs2*, *Tnf*, *Zfp36*, and *Zfp36l2* (Figures 4E and S3A). The fold change between BMDM^WT^ and BMDM^TLR4^ was greatest in injectisome-mutant infection (Figure S4C); however, WT *Salmonella* infection of WT cells gave rise to maximal TE (Figure 4E). This indicates that the SPI-1 injectisome plays a key role in the translational upregulation of these genes, alongside TLR4. *K*-mer enrichment analysis of the 3′ UTR sequences of these genes revealed multiple copies of a 6-nt-long AU-rich motif (UAUUUA) at multiple loci in the 3′ UTRs of all transcripts (Figures S4D and S4E), aligning with previous reports on LPS-driven translational reprogramming through the modulation of AU-rich RNA-binding proteins.^4^ Applying the same selection criteria to look for genes with TLR4-dependently downregulated TE (log_2_FC < −1) did not identify any transcripts, likely reflecting the stringency of the selection criteria, requiring differential expression across multiple conditions during the first hour of infection.

Six of the translationally upregulated genes showing TLR4-dependent translational upregulation are directly involved in cell-to-cell signaling, as cytokines and generators of signaling messengers, with the others also having key roles in modulating cell signaling pathways (Figure 4D). These genes were further divided into three clusters by their translational patterns throughout infection (Figure 4F): genes that exhibit rapid TLR4- and injectisome-dependent concurrent translational and transcriptional induction over time (cluster 1); genes that exhibited rapid TLR4-dependent, injectisome-independent translational induction with enhanced TE over time with little transcriptional response (cluster 2); and genes that exhibit rapid, but smaller, TLR4-dependent and injectisome-independent translational induction in response to infection with little transcriptional change (cluster 3). Due to the high-resolution nature of the Ribo-seq dataset, translation of these genes could be directly visualized at the sub-codon level (Figure 5). Here, it is again clear that protein synthesis is not always linearly correlated with mRNA abundance and that, while *Salmonella* with an intact SPI-1 injectisome is able to limit the TLR4-dependent transcriptional response, it does not repress the translational response. Rather, it may act synergistically to further increase TE of these induced genes.

**Figure 5:**
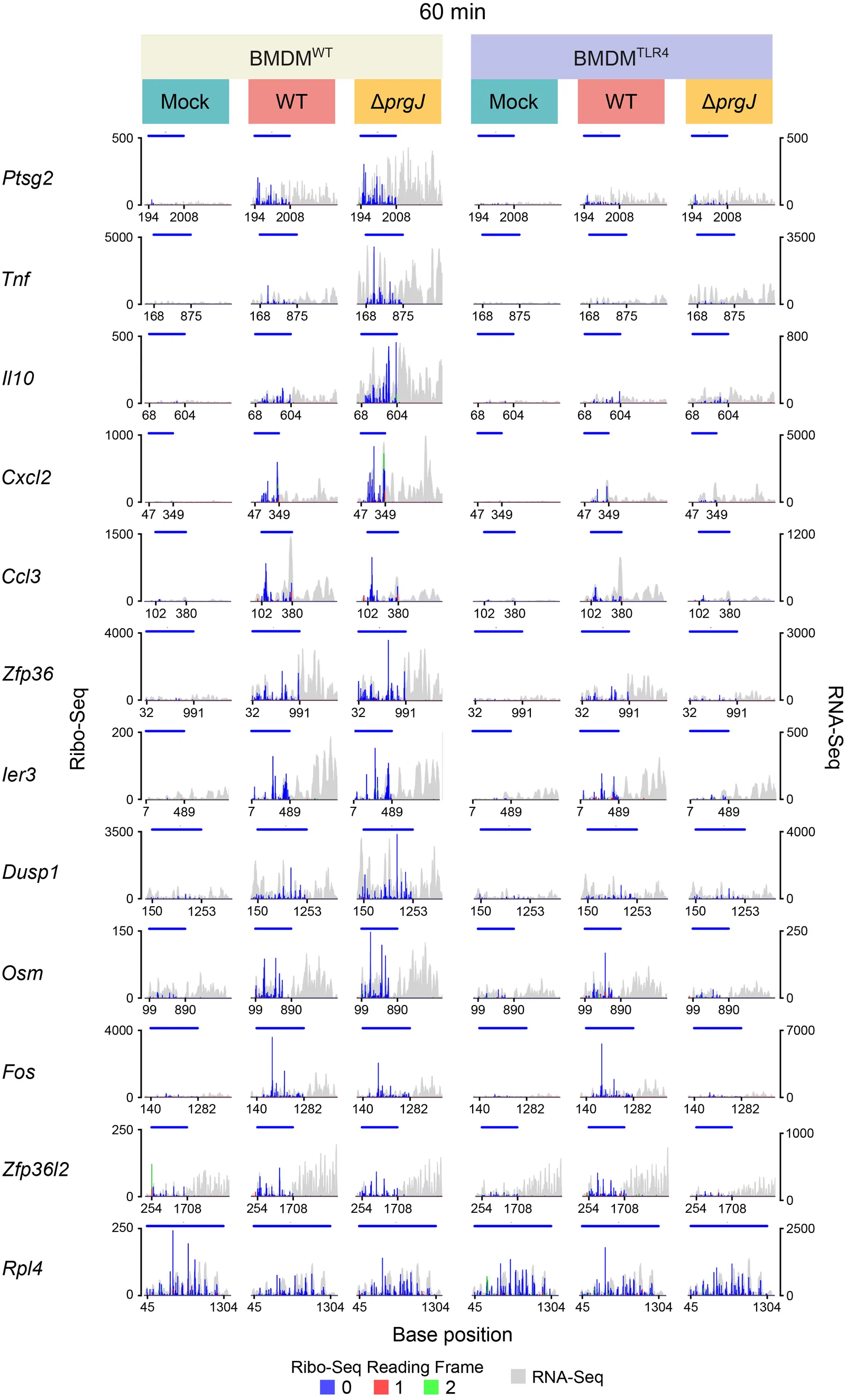
Ribo-seq read map showing inferred P-site position of ribosomes on the indicated genes and coverage of RNA-seq reads of samples at 60 min post infection Bars are colored by the reading frame of the inferred P-site position relative to the gene’s start codon (frame 0; blue). Reads in green and red align out of frame. *Rpl4* is shown as an example gene expressed consistently in all conditions.

To determine if the observed increases in TE led to an increase in protein abundance, western blotting, and quantitative reverse-transcription PCR (RT-qPCR) were performed at 1 h post infection of WT and TLR4-deficient iBMDMs. *Tnf* was chosen as a candidate exhibiting dramatic TLR4-dependent transcriptional and translational upregulation, whereas *Fos* was chosen because its upregulation is predominantly driven by increased translation with much smaller changes in mRNA abundance. The relative abundance of these transcripts measured by RT-qPCR closely matched the RNA-seq analysis (Figures S5A and 4F). Both candidates showed significantly greater induction during the infection of WT over TLR4-deficient host macrophages, with the abundance of *Tnf* being greatest in injectisome-mutant infection of WT macrophages. At the protein level, TNF-α was only detected in the injectisome-mutant *Salmonella* infection of WT iBMDMs (Figures S5B and S5C), which mirrors the condition exhibiting maximal expression in the Ribo-seq data (Figures 4F and 5). For c-FOS, infection of WT host macrophages yielded significantly more protein than in TLR4-deficient macrophages for both bacterial strains (Figures S5B and S5C). However, while the Ribo-seq data indicated similar *Fos* translation in WT *Salmonella* infection of WT and TLR4-deficient macrophages (Figures 4F and 5), c-FOS protein abundance actually appeared downregulated in WT-infected TLR4-deficient iBMDMs. This suggests that c-FOS protein abundance may be subject to specific post-translational regulation in addition to the regulation of its synthesis that we identified.

## Discussion

Previous reports have demonstrated that both *Salmonella*^29^^,^^30^ and purified LPS^27^ can trigger actin ruffling in stimulated macrophages (Figure 1). Our results support these as two independent processes, i.e., the *Salmonella* trigger mechanism and LPS acting on TLR4, and demonstrate they both occur rapidly, within 15 min of exposure. Activation of other cell surface TLRs did not result in an increase in ruffling, consistently with previous reports that LPS-induced macrophage actin ruffling is a MyD88-independent process.^44^^,^^45^ This aligns with a recent report detailing the distinct pathways activated by *Salmonella* SPI-1 effectors leading to membrane ruffling and how TLR4-driven factors modulate it.^35^ In that study, they identified a TLR4-PIEZO1-TMEM16F signaling axis that is important for the resolution of membrane ruffles during *Salmonella* invasion. This is consistent with our observation that there is a greater amount of ruffling upon WT *Salmonella* infection in TLR4^−/−^ macrophages compared to WT macrophages.

During the first 15–60-min window of *Salmonella* infection, the transcriptional response is, as anticipated, minimal (Figure 2B). The few genes identified as being differentially regulated at the transcriptional level are associated with immune processes (Figure 2C), consistently with prior studies on the transcriptional response to TLR4 stimulation by LPS.^20^^,^^24^ In contrast to the limited transcriptional response, a more robust translational response was observed during both WT and SPI-1 injectisome-deficient *Salmonella* infection (Figures 3A, 3B, and S3). This heightened translational activity is driven by the modulation of both pre-existing and newly synthesized mRNAs in the cytoplasm and is consistent with findings from our previous study on *Salmonella*-induced translational regulation.^23^ Notably, much of this rapid translational response was absent when both the bacterial SPI-1 injectisome and host TLR4 were missing, highlighting their critical role in orchestrating translational responses during the first hour of *Salmonella* infection of macrophages (Figure 4C). Genes identified as upregulated at the levels of RNA abundance and protein synthesis showed enrichment for roles in the immune response, stress response, and cell signaling (Figures 2C and 3C). Notably, enrichment for cell death-related GO terms was only seen in injectisome-mutant *Salmonella* infection, where host cell death is minimal. More genes were identified as TLR4-dependently transcriptionally upregulated in infection with SPI-1 injectisome-deficient *Salmonella* than WT. This was also true for genes with enhanced protein synthesis. Many of these upregulated genes still showed upregulation in WT *Salmonella* infection, though to a lesser degree than in injectisome-mutant *Salmonella* infection and so did not meet our selection criteria. Changes in transcription and protein synthesis were found to be non-linearly correlated, highlighting the role of altered TE in regulating expression from a given transcript (Figure 3D).

Of the genes identified as translationally regulated, 6 out of 11 had direct roles in cell-to-cell signaling, with the rest also involved in modulating signaling pathways (Figure 4D). This includes genes encoding the pro-inflammatory cytokines TNF, CXCL2, and CCL3, and the enzyme PTGS2 (also called COX2), which produces pro-inflammatory prostaglandins. This response may be supported by the rapid expression of the c-FOS transcription factor, the stress-survival factor IER3, and the pleiotropic cytokine OSM. Alongside this pro-inflammatory axis, however, there is simultaneous upregulation of the MAPK/JNK phosphatase DUSP1,^46^ the AU-rich RNA-destabilizing proteins ZFP36 (also called Tristetraprolin, TTP) and ZFP36L2,^47^ and the anti-inflammatory cytokine IL-10. The concurrent translation of these apparent resolution factors suggests that while TLR4-induced translational upregulation results in a rapid burst of inflammatory mediators, negative feedback mechanisms are engaged to ensure it is self-limiting, as has previously been reported.^4^

Interestingly, the TE of these genes in WT *Salmonella* infection exceeded that in injectisome-mutant *Salmonella* infection, despite lower transcript abundance (Figures 4E and 4F). It is therefore clear that the *Salmonella* SPI-1 injectisome plays an important role in attenuating the TLR4-dependent transcriptional response, likely due to SPI-1 effector-dependent modulation of signaling downstream of TLR4 as has previously been described.^6^^,^^7^^,^^8^^,^^9^^,^^10^^,^^11^ However, this inhibition appears to occur downstream of a branch in the signaling pathway that governs TLR4-dependent translational regulation, as the translational response is not inhibited. Rather, the translational response is maximal with intact SPI-1 injectisome, suggesting that it in some way augments the TLR4-dependent increases in translation of these transcripts. This may be due to the action of effectors secreted into the host cell through the SPI-1 injectisome or as a response to injectisome-mediated host membrane injury as has previously been demonstrated for other host translational responses.^23^

The swift synthesis of cytokines is of particular importance in *Salmonella* infection given the rapid induction of SPI-1 injectisome-dependent host cell death (Figure S1A), leaving only a short window for most infected macrophages to mount an immune response. Modulation of the TE of cytokine-encoding mRNAs in parallel with increasing their abundance allows for a more robust response. This is particularly important where the transcriptional arm of the host response is antagonized by bacterial effectors.

This prioritization of translational control likely represents a general, fast-acting host defense strategy applicable to responses mediated by other pattern recognition receptors. Many different bacterial pathogens employ effectors to target host transcriptional responses.^48^^,^^49^ Understanding how host translational control persists or adapts in the face of this common bacterial strategy is thus crucial for identifying the regulatory networks that ultimately govern the outcome of infection.

### Limitations of the study

This study is intentionally focused on the first 60 min of infection, the key window in which the dominant translational response was previously observed,^23^ and therefore it does not yet address how the TLR4-dependent translational program may evolve at later stages as broader transcriptional responses emerge and *Salmonella* transitions toward SPI-2-associated intracellular remodeling. In addition, bulk profiling provides a robust population-level view of early events but may obscure heterogeneity between infected macrophages and uninfected bystander cells. The specific SPI-1 effector(s) responsible for transcriptional inhibition, and their mechanisms of action, remain to be identified. Addressing these points through extended infection time courses, single-cell approaches, and effector-level mechanistic dissection represents the next step enabled by this framework. Finally, this study utilizes the non-typhoidal *Salmonella* (NTS) serovar Typhimurium strain SL1344. Validation in other NTS serovars would be required to determine if these responses are broadly applicable to other *Salmonella* infections, given their large pathogenic and metabolic diversity.

## Resource availability

### Lead contact

Requests for further information and resources should be directed to and will be fulfilled by the lead contact, Betty Y.-W. Chung (bcy23@cam.ac.uk).

### Materials availability

This study did not generate new unique reagents.

### Data and code availability

• Raw sequencing data are available from ArrayExpress: E-MTAB-14159. Tables of calculated fold changes in gene expression at the levels of mRNA abundance, protein synthesis, and TE are presented in Table S5.
• This study did not generate original code; however, customized scripts are available from the lead contact.
• Any additional information required is available upon request to the lead contact.

## Acknowledgments

G.W. was supported by a 10.13039/100020950Department of Pathology, University of Cambridge, United Kingdom PhD studentship, and F.L. was supported by a 10.13039/501100000268BBSRC DTP studentship. P.T. and C.E.B. were supported by the 10.13039/100010269Wellcome Trust (108045/Z/15/Z), B.Y.-W.C., R.J., G.W., F.L., and M.P.B. were supported by a 10.13039/501100000265Medical Research Council fellowship and 10.13039/501100000268BBSRC project grants to B.Y.-W.C. [MR/R021821/1, BB/X001261/1, BB/V017780/1 and BB/V006096/1].

## Author contributions

B.Y.-W.C., and C.E.B. conceived the research; B.Y.-W.C., C.E.B., G.W., and R.J., designed experiments;. R.J. and G.W. performed molecular and cell biology experiments; R.J., M.P.B., G.W., and F.L. performed bioinformatics; P.T. maintained mouse colonies and processed mice;. B.Y.-W.C. and G.W. wrote the manuscript.

## Declaration of interests

The authors declare no competing interests.

## STAR★Methods

### Key resources table

**Table:** 

REAGENT or RESOURCE	SOURCE	IDENTIFIER
**Antibodies**		

Rabbit anti-TNF-α	Abcam	Cat#ab9739; RRID: AB_308774
Rabbit anti-*c*-Fos	Cell Signaling	Cat#2250; RRID: AB_2247211
Anti-rabbit conjugated to HRP	Cell Signaling	Cat#7074; RRID: AB_2099233
Goat anti-*Salmonella* CSA	Insight Biotechnology	Cat#5310-0322; RRID: AB_2890923
Anti-goat IgG conjugated to Alexa 488	ThermoFisher	Cat#A-11055; RRID: AB_2534102

**Bacterial and virus strains**		

*Salmonella enterica* sv. Typhimurium SL1344 WT	National Collection of Type Cultures (NCTC)	NCTC 13347
*Salmonella enterica* sv. Typhimurium SL1344 Δ*prgJ*	Man et al.^31^	N/A

**Chemicals, peptides, and recombinant proteins**		

Fluoresbrite Yellow Green Microspheres 1.00 μm	Polysciences	Cat#17154
Gentamicin sulfate	Melford	Cat#G38000
*Escherichia coli* O127:B8 LPS	Sigma-Aldrich	Cat#L3129
Recombinant *S*. Typhimurium flagellin	Abcam	Cat#ab201366
Pam3CSK4	Tocris	Cat#4633
Phalloidin-CF594 conjugate	Biotium	Cat#00045
DAPI	Invitrogen	Cat#62248
M-CSF	Peprotech	Cat#315-02
cOmplete Mini	Roche	Cat#11836153001
PhosSTOP	Roche	Cat#4906845001
TRIzol	Invitrogen	Cat#15596026

**Critical commercial assays**		

CytoTox96 Non-radioactive Cytotoxicity Assay kit	Promega	Cat#G1780
Mouse IL-1 beta/IL-1F2 DuoSet ELISA kit	R&D Systems	Cat#DY401
SuperSignal West Pico PLUS substrate	Thermo Scientific	Cat#34580
M-MLV reverse transcriptase	Promega	Cat#M1701
iTaq Universal SYBR Green Supermix	Bio-Rad	Cat#1725121

**Deposited data**		

Raw sequencing data	This paper	ArrayExpress: E-MTAB-14159

**Experimental models: Cell lines**		

Immortalized murine bone marrow derived macrophages (iBMDM) WT	BEI Resources, NIAID, NIH	NR-9456
Immortalized murine bone marrow derived macrophages (iBMDM) TLR4^−/−^	BEI Resources, NIAID, NIH	NR-9458

**Experimental models: Organisms/strains**		

Mouse: C57BL/6J WT	Charles River Laboratories	C57BL/6J WT
Mouse: C57BL/6J TLR4^−/−^	Hoshino et al.^50^	C57BL/6J TLR4^−/−^

**Oligonucleotides**		

qPCR primer *Supt16* Fwd: GTACTACCGGCGAGTGAAGA	This paper	N/A
qPCR primer *Supt16* Rev: ACCAAAGAGCCACGTCTGT	This paper	N/A
qPCR primer *Tnf* Fwd: AGGCACTCCCCCAAAAGATG	This paper	N/A
qPCR primer *Tnf* Rev: CATTTGGGAACTTCTCATCCC	This paper	N/A
qPCR primer *Fos* Fwd: GGTTTCAACGCCGACTACGA	This paper	N/A
qPCR primer *Fos* Rev: CTGCGCAAAAGTCCTGTGTG	This paper	N/A

**Software and algorithms**		

fastx_clipper	Hannon^51^	http://hannonlab.cshl.edu/fastx_toolkit/
Bowtie	Langmead et al.^52^	https://bowtie-bio.sourceforge.net/index.shtml
riboSeqR	Chung et al.^53^	https://bioconductor.org/packages/release/bioc/html/riboSeqR.html
edgeR	Robinson et al.^54^	https://bioconductor.org/packages/release/bioc/html/edgeR.html
Xtail	Xiao et al.^55^	https://github.com/xryanglab/xtail
gProfiler2	Kolberg et al.^56^	https://cran.r-project.org/web/packages/gprofiler2/
Sylamer	van Dongen et al.^57^	https://github.com/micans/sylamer
ComplexHeatmap	Gu et al.^58^	https://bioconductor.org/packages/release/bioc/html/ComplexHeatmap.html
Image Studio 6.1	LI-COR Biosciences	https://www.licorbio.com/image-studio
Design and Analysis 2	Thermo Fisher Scientific	https://www.thermofisher.com/uk/en/home/technical-resources/software-downloads/quantstudio-6-7-pro-real-time-pcr-system.html

### Experimental model and study participant details

#### Mice

All mice were maintained in a specific pathogen-free facility according to the Animals Scientific Procedures outlined by the UK Home Office regulations. All work involving live animals complied with the University of Cambridge Ethics Committee regulations and was performed under the Home Office Project License numbers 80/2572 and P48B8DA35.

#### Primary cells and cell lines

Primary BMDMs were isolated and differentiated as previously described^31^ from male 8–12-week-old WT or TLR4^−/−^ C57BL/6 mice. iBMDM were previously generated by viral transformation of BMDMs from these mice as previously described^59^ and obtained from BEI Resources. All cells were routinely grown in high-glucose DMEM supplemented with 10% fetal bovine serum at 37 °C and 5% CO_2_. Medium for primary BMDM was supplemented with 20 ng/mL M-CSF (Peprotech). Cell lines were verified by morphological assessment and comparison of phenotypic responses to *Salmonella* and LPS with published data. Cell lines were routinely checked for mycoplasma contamination.

### Method details

#### *Salmonella* infection of macrophages

Cells were infected at a multiplicity of infection of 10 CFU/cell with WT or Δ*prgJ S*. Typhimurium SL1344 as previously described.^23^ Briefly, bacteria were grown in LB broth to late exponential phase (OD_600_ = 2) at 37 °C and shaking at 200 rpm. They were then washed in cell culture medium and added to the cultured cells. At 15 min post infection, gentamicin (50 μg/mL final concentration for primary BMDM, 100 μg/mL final concentration for all others) was added to ensure synchronous infection.

#### Immunofluorescence microscopy

Cells were plated onto glass coverslips and grown until 50% confluency before being infected or stimulated as indicated. 1 μm Fluoresbrite Yellow Green Microspheres (Polysciences) were washed in cell culture medium and used as a control for phagocytosis at 100 beads per cell. TLR4 was stimulated with *Escherichia coli* O127:B8 LPS (Sigma-Aldrich, #L3129) at 500 ng/mL. TLR5 was stimulated with recombinant *S*. Typhimurium flagellin (Abcam, #ab201366) at 40 ng/mL. TLR1/2 was stimulated with Pam3CSK4 (Tocris, #4633) at 500 ng/mL. Mock stimulations were conducted with vehicle only: water for LPS and flagellin; 50% ethanol for Pam3CSK4; medium only for beads and infections.

Cells were fixed with 4% paraformaldehyde and stained with Phalloidin-CF594 conjugate (Biotium), DAPI (Invitrogen), and goat anti-CSA (Insight Biotechnology) followed by donkey anti-goat IgG conjugated to Alexa 488 (ThermoFisher). Cells were mounted onto slides and imaged at 100× magnification. 20 random fields were captured per replicate and cells were classified as ruffled or not as described previously.^27^ 50 cells were chosen at random to represent each replicate. A one-sided Student’s t test was used to assess the significance of differences.

#### Ribosome profiling and RNA sequencing

Combined ribosome profiling and RNA sequencing of infected macrophages was conducted as previously described.^23^ Briefly, culture supernatant was removed from cells, and they were flash frozen. Cells were lysed in buffer containing cycloheximide and chloramphenicol, and lysates were split for parallel RNA-Seq and Ribo-Seq. For Ribo-Seq, lysates were treated with RNase I and fragments protected from digestion by the ribosome were purified. For RNA-Seq, total cellular RNA was fragmented by alkaline hydrolysis. This was followed by library generation as previously described.^53^^,^^60^^,^^61^ Sequencing was performed using NextSeq-500 (Illumina).

#### Cytotoxicity assays

Cells were plated in 24-well plates and grown until 70–80% confluency before being infected as indicated. Cytotoxicity was measured by lactate dehydrogenase (LDH) release using CytoTox 96 Non-radioactive Cytotoxicity Assay kit (Promega) according to the manufacturer’s instructions. Values are relative to total host cell lysis in 0.9% Triton X-100.

#### ELISA

Culture supernatants were removed from infected cells at the indicated timepoint. ELISAs were performed to quantify IL-1β in these culture supernatants using the Mouse IL-1 beta/IL-1F2 DuoSet ELISA kit (R&D Systems) per the manufacturer’s instructions.

#### Polysome profiling

Polysome profiling has been described previously.^62^ Briefly, cell lysates were prepared as for ribosome profiling and then centrifuged over a linear 10–45% sucrose gradient prepared using a Gradient Master (BioComp). RNA concentration, measured by absorbance at 254 nm, was then traced across the gradient using a Density Gradient Fractionation System (Brandel). Traces were aligned and scaled by normalising to the height of the monosome peak, allowing direct visual assessment of changes in the relative abundance of polysomes between conditions.

#### Western immunoblotting

Protein was harvested from PBS-washed cell monolayers disrupted with lysis buffer (50 mM Tris-HCl pH 8.0, 150 mM NaCl, 1 mM EDTA, 10% glycerol, 1% Triton X-100, 0.1% IGEPAL-CA630) that contained protease (cOmplete Mini, Roche) and phosphatase inhibitors (PhosSTOP, Roche). Protein was separated by SDS-PAGE and transferred to a nitrocellulose membrane. Prior to blocking in 5% non-fat dairy milk, membranes were stained with 0.1% Ponceau S in 5% acetic acid (Sigma). Membranes were blotted with rabbit anti-TNF-α (Abcam, #ab9739) and rabbit anti-*c*-Fos (Cell Signaling, #2250) primary antibodies and then anti-rabbit conjugated to HRP (Cell Signaling, #7074). HRP was assayed using SuperSignal West Pico PLUS substrate (Thermo Scientific). Signals were captured using an Azure 500Q imager (Azure Biosystems).

#### RT-qPCR

RNA was extracted from cells using TRIzol (Invitrogen) per the manufacturer’s instructions. Reverse transcription was performed using M-MLV reverse transcriptase (Promega) with random hexamer primers per the manufacturer’s instructions. Real-time qPCR was performed using iTaq Universal SYBR Green Supermix (Bio-Rad) and assayed on a QuantStudio 6 Pro system (Applied Biosystems) per the manufacturer’s instructions. Primer pairs used for qPCR were designed so that at least one spanned an exon-exon junction.

### Quantification and statistical analysis

#### Sequencing data analysis

Reads from two replicate infections were combined and trimmed to remove adaptors using fastx_clipper from the FASTX Toolkit.^51^ Trimmed reads of at least 15 nt in length were then aligned sequentially to mouse rRNA and mouse mRNA using Bowtie.^52^ Mouse reference sequences were based on release mm10 (Figures S6A and S6B). Counting was performed using riboSeqR,^53^ with only reads 28–32 nt in length and aligning within the predominant reading frame counted for Ribo-Seq reads (Figure S6C). Normalisation was performed with edgeR^54^; TE was calculated as the ratio of normalised Ribo-Seq reads to RNA-Seq reads. Differential expression analysis was performed using Xtail.^55^ *p* values were estimated using the two-tailed Bayesian framework from Xtail, comparing mRNA and RPF log_2_FC and log_2_ ratios across two pipelines and reporting the more conservative result with Benjamini–Hochberg correction for multiple comparisons. Genes with corrected *p* values less than 0.05 were considered to have significantly different translational efficiency. GO term enrichment was performed using g:Profiler^56^ and similar GO terms were manually grouped for plotting. For individual gene plots, only Ribo-Seq reads of 28 and 29 nt in length were plotted and the corresponding ribosome P-site positions were inferred from the metagene translatome (Figure S6D). Heatmaps were produced with ComplexHeatmap.^58^ *K*-mer enrichment analysis was performed using Sylamer.^57^

#### Western blot quantification

For quantifying relative protein abundance, western blot and Ponceau S stain signals were quantified by densitometry using the Image Studio software package (LI-COR Biosciences). The specific western blot signal was normalized to the total protein abundance for the corresponding lane. A one-sided Welch’s *t* test was used to assess the significance of differences.

#### RT-qPCR quantification

The Design & Analysis 2 software package (Thermo Fisher Scientific) was used to determine C_t_ values. Relative gene expression was calculated using the ΔΔC_t_ method by first normalising the C_t_ values of the genes of interest to the housekeeping gene *Supt16* (ΔC_t_) and then normalising to the mean ΔC_t_ of the reference sample (mock-infected WT macrophages). A one-sided Welch’s *t* test was used to assess the significance of differences.

#### Statistical analysis

All statistical analyses were performed in R. The specific statistical tests utilised for each experiment, including adjustments for multiple comparisons, are reported in figure legends or detailed above, where *n* represents the number of biological replicates. Statistical significance was defined as an adjusted *p* value <0.05. Unless otherwise stated, error bars represent standard error of the mean. Where adjusted *p* values are represented graphically, n.s. indicates adjusted *p* value >0.05; ∗ indicates adjusted *p* value <0.05 and ∗∗ indicates adjusted *p* value <0.01.

## Supplemental information

Document S1. Figures S1–S6

Table S1. Enriched GO terms of genes with upregulated RNA abundance in WT *Salmonella* infection of BMDM^WT^ over BMDM^TLR4^

Table S2. Enriched GO terms of genes with upregulated RNA abundance in Δ*prgJ Salmonella* infection of BMDM^WT^ over BMDM^TLR4^

Table S3. Enriched GO terms of genes with upregulated RNA abundance in WT *Salmonella* infection of BMDM^WT^ over BMDM^TLR4^

Table S4. Enriched GO terms of genes with upregulated RNA abundance in Δ*prgJ Salmonella* infection of BMDM^WT^ over BMDM^TLR4^

Table S5. Fold changes in gene expression calculated in this study
