# Supplementary material for: Toll-like receptor 4-driven translational reprogramming in macrophages is unaffected by Salmonella SPI-1 activity
Source: iScience. 2026 Oct 26;29(11):117901. doi: 10.1016/j.isci.2026.117901 (PMC13734781; doi:10.1016/j.isci.2026.117901)
Supplement: Supplementary file 1 [file mmc1.pdf]

iScience, Volume *29*

Supplemental information

Toll-like receptor 4-driven translational reprogramming in macrophages is unaffected by *Salmonella* SPI-1 activity

George Wood, Rebecca Johnson, Matthew P. Brember, Filip Lastovka, Panagiotis Tourlomousis, Clare E. Bryant, and Betty Y.-W. Chung

Figure S1

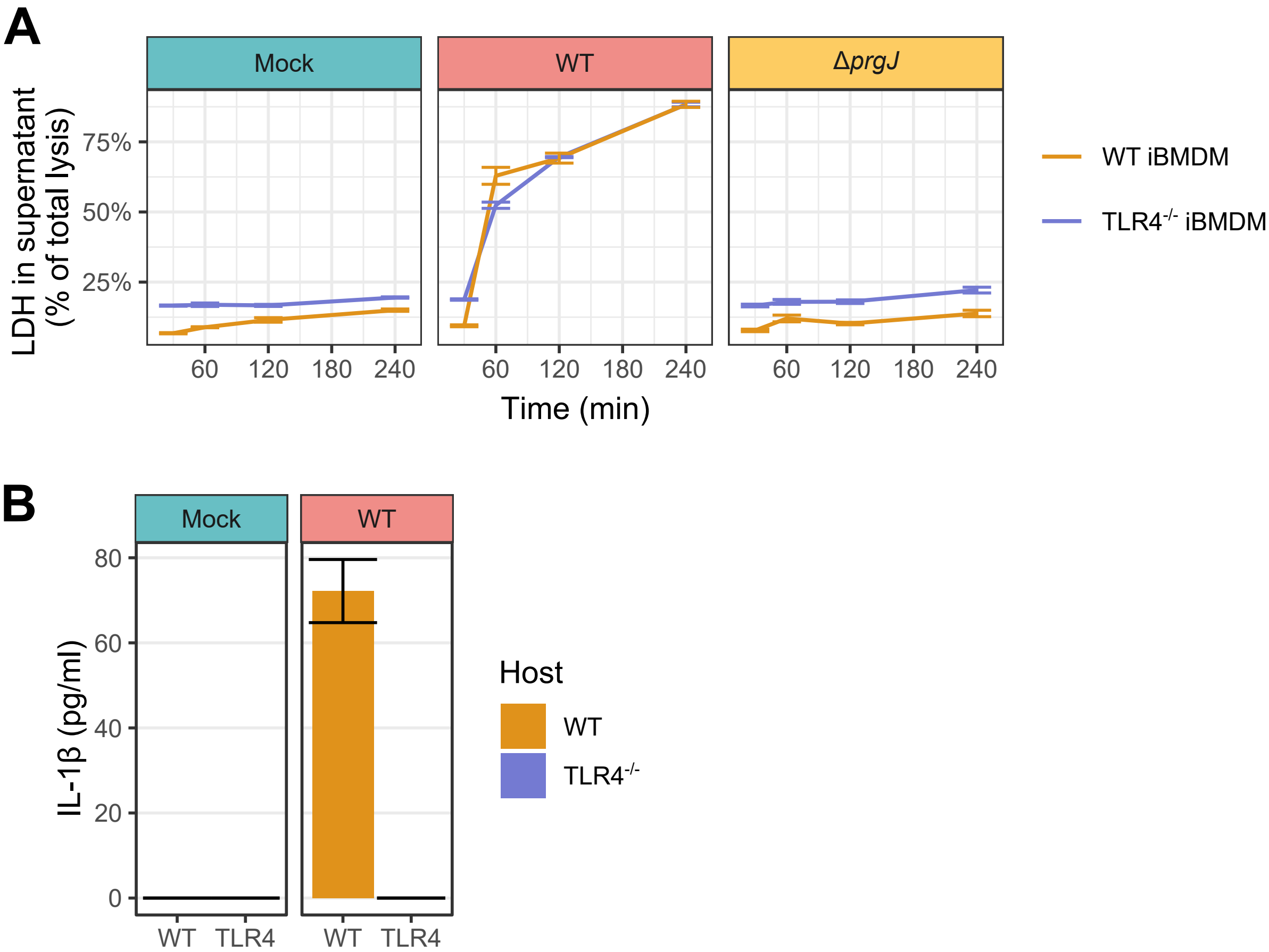

Figure S1: TLR4- and SPI-1-dependent host cytotoxicity and IL-1β release during *Salmonella* infection. (**A**) Cytotoxicity of WT or Δ*prgJ Salmonella* infection in WT and TLR4$^{-/-}$ iBMDMs as measured by LDH release into culture supernatant. Values are relative to those following total cell lysis. *n*=2, data representative of 2 independent experiments. (**B**) IL-1β in culture supernatant 4 hours after WT *Salmonella* infection of WT and TLR4$^{-/-}$ iBMDMs as determined by ELISA. *n*=3. (**A** and **B**) Data are represented as mean ± SEM.

Figure S2

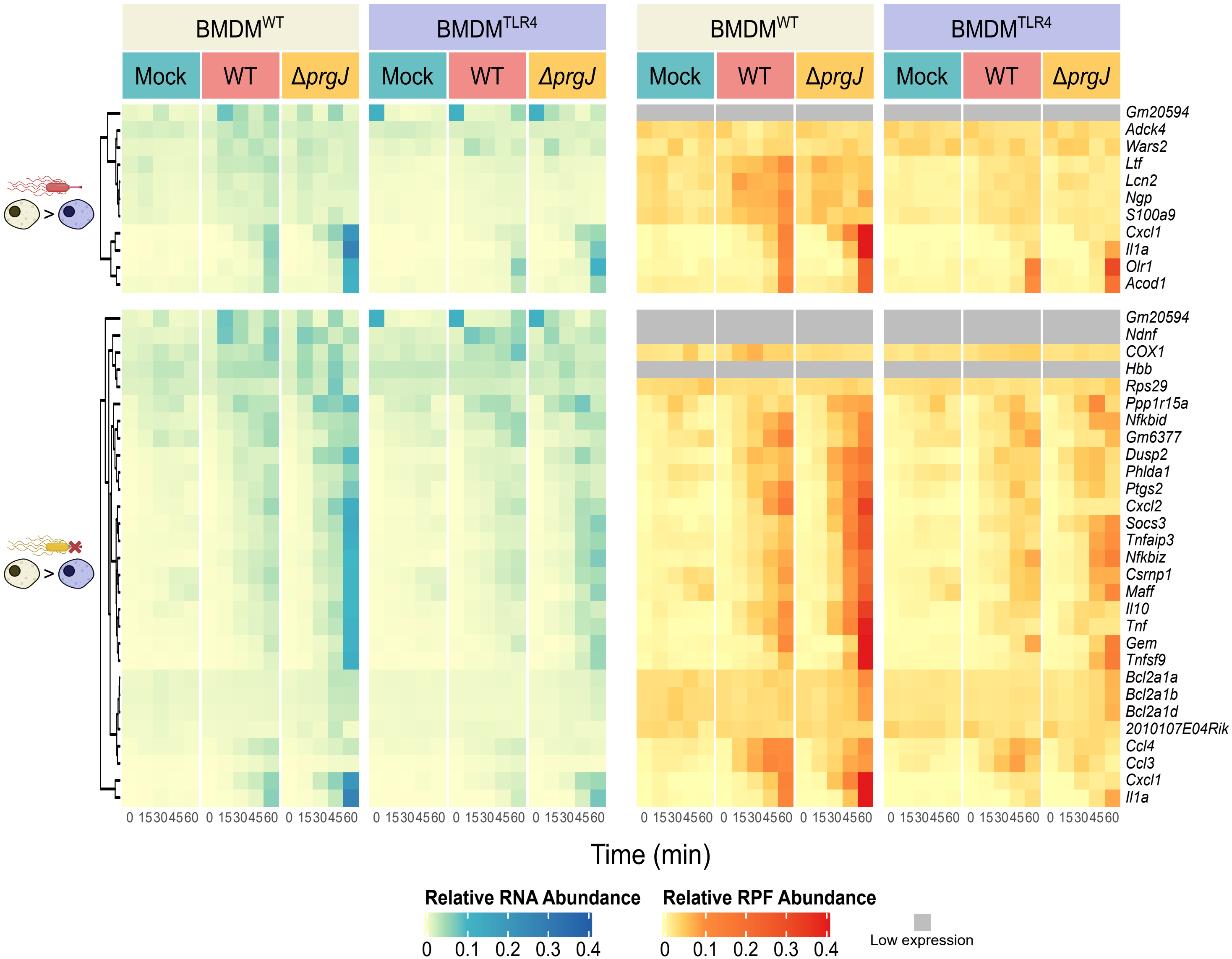

Figure S2: Expression of genes transcriptionally upregulated in a TLR4-dependent manner over *Salmonella* infection (WT: top, Δ*prgJ*: bottom) at the levels of both transcript abundance and protein synthesis. Genes are clustered by RNA abundance. Values are relative to total normalised RNA- and Ribo-Seq counts of that gene.

Figure S3

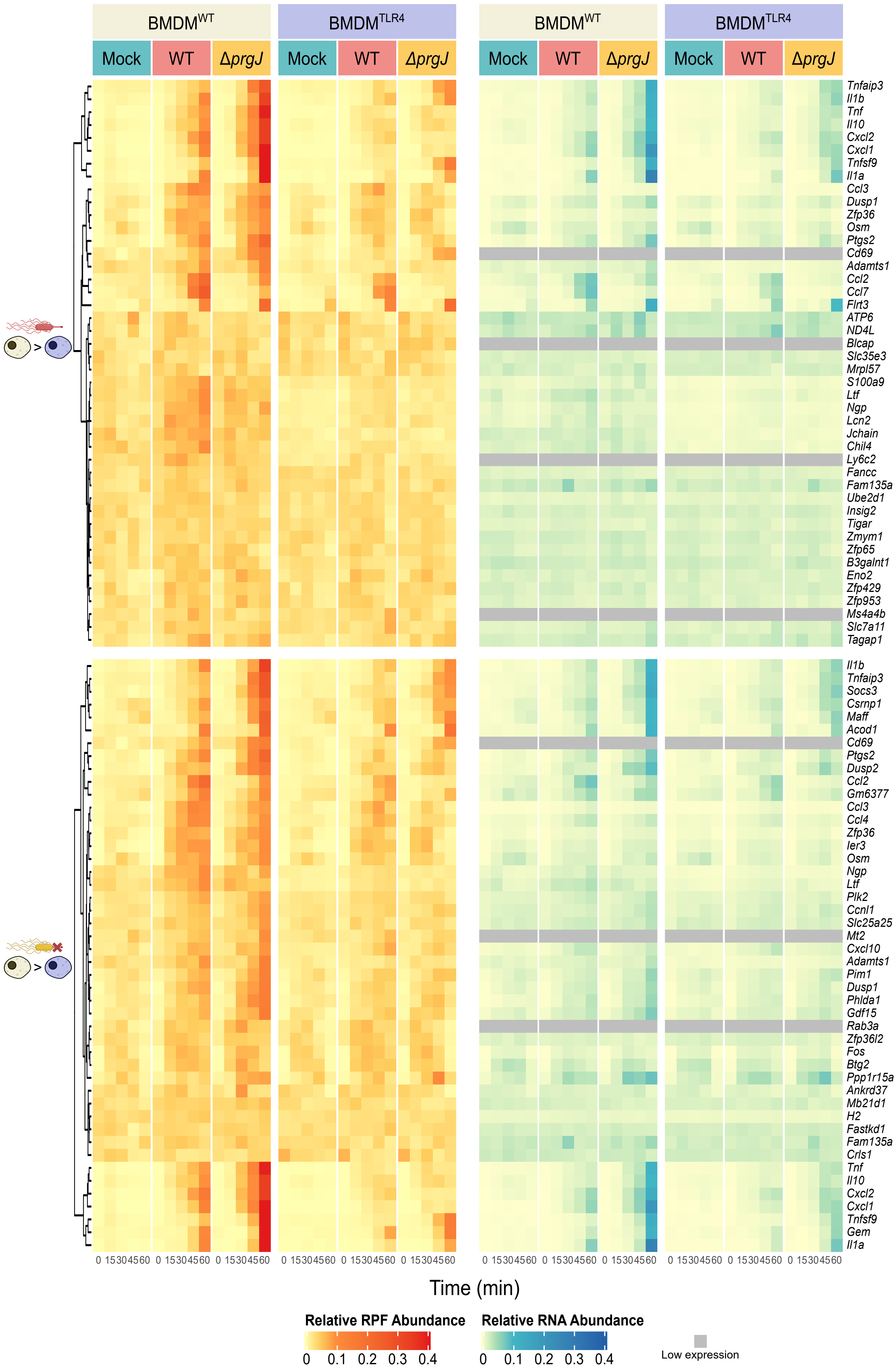

Figure S3: Expression of genes with upregulated protein synthesis in a TLR4-dependent manner over *Salmonella* infection (WT: top, Δ*prgJ*: bottom) at the levels of both transcript abundance and protein synthesis. Genes are clustered by amount of protein synthesis. Values are relative to total normalised RNA- and Ribo-Seq counts of that gene.

Figure S4

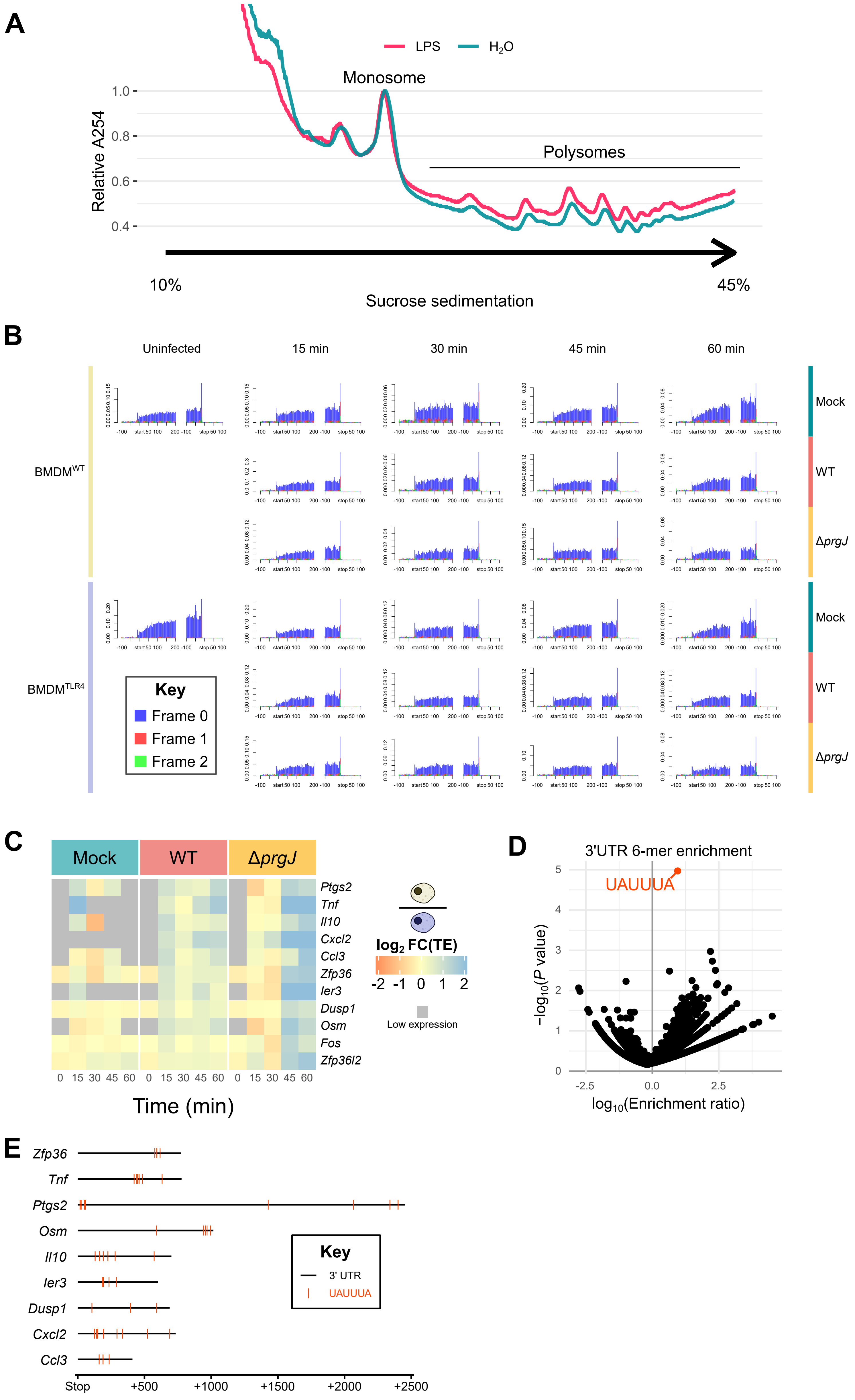

Figure S4: Ribosome profiling and polysome analysis reveal TLR4-dependent translational upregulation. (**A**) Polysome profile trace of WT iBMDMs stimulated with 500 ng/ml LPS or water vehicle for 60 min. Traces are scaled to equalise their monosome peak. Representative of 2 independent experiments. (**B**) Meta-gene translatome from ribosome profiling of *Salmonella* infected $BMDM^{WT}$ and $BMDM^{TLR4}$ over the first 60 min of infection. Histograms show ribosome protected fragment 5′ ends relative to start and stop codons coloured by their reading frame relative to the first codon of the coding sequence. (**C**) Heatmap showing the log_2FC of translation efficiency (TE) in infected $BMDM^{WT}$ over $BMDM^{TLR4}$ for genes with significantly upregulated TE. An expression cut-off of 10 reads was used for both RNA and protein synthesis, below which TE could not be calculated reliably. (**D**) 6-mer enrichment analysis on the 3′ UTRs of translationally upregulated transcripts. Significance was determined with Sylamer[50] using the hypergeometric distribution. (**E**) Distribution of the enriched UAUUUA motif within the 3′ UTRs of the translationally upregulated transcripts represented by vertical red bars. The horizontal black line represents the full 3′ UTR length of that transcript.

Figure S5

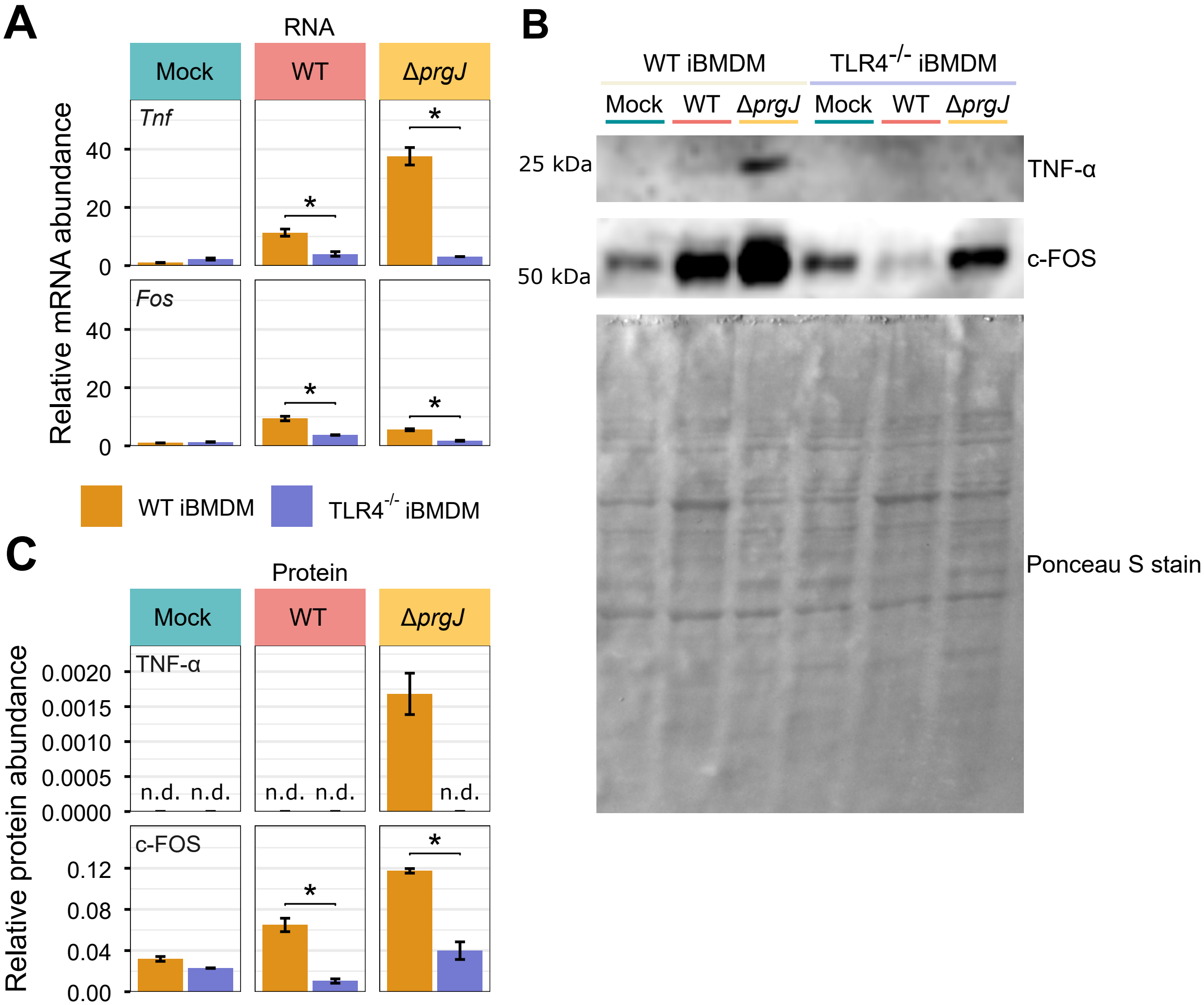

Figure S5: Protein products of translationally upregulated transcripts increase in abundance. (**A**) Quantification of *Tnf* and *Fos* mRNA abundance by RT-qPCR. Values shown are relative to mock infection of WT iBMDM and normalised to the housekeeping gene *Supt16*; $n = 3$. (**B**) Protein abundance at 60 min post WT and injectisome-mutant *Salmonella* infection of WT and TLR4-deficient iBMDM. (Top) Western blots against TNF-α and c-FOS. (Bottom) Total protein stained with Ponceau S. Representative of 2 independent experiments. (**C**) Quantification of protein abundance from western blots as in **B**, normalised to total protein as measured by Ponceau S stain. N.d. indicates the protein was not detected; $n = 2$. (**A** and **C**) *P* values determined by Benjamini–Hochberg adjusted one-sided Welch's *t*-test; * indicates adjusted *P* value < 0.05. Data are represented as mean ± SEM.

Figure S6

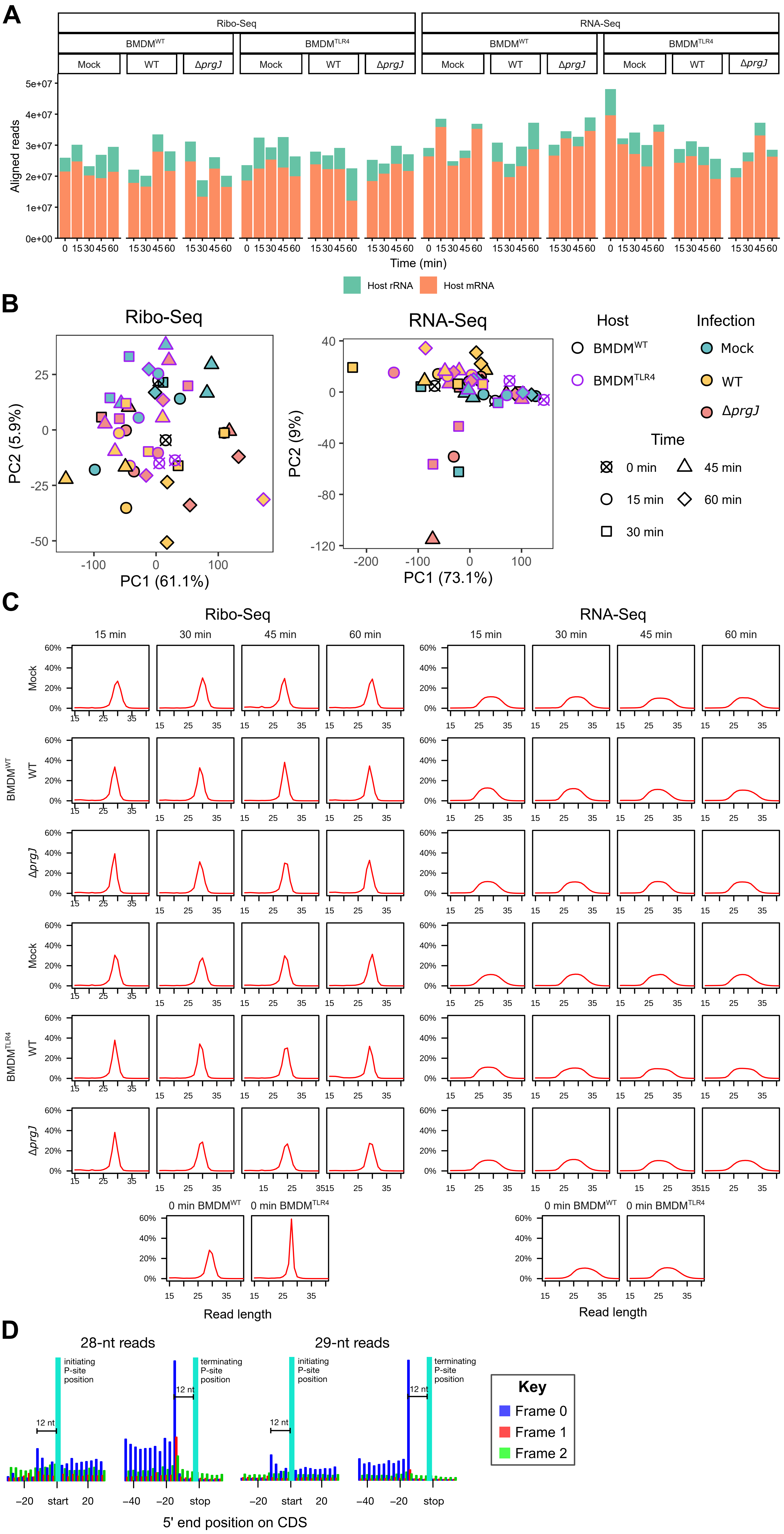

Figure S6: Summary of Ribo-Seq and RNA-Seq datasets. (**A**) Number of reads aligning to host mRNA and host rRNA for Ribo-Seq and RNA-Seq at each condition and time point. (**B**) Principal component analysis of log_2 scaled and centred individual replicate raw counts. Points are coloured by host cell (outline) and infection condition (inner fill), and their shape indicates their timepoint. (**C**) Size distribution of Ribo-Seq and RNA-Seq reads aligning to mRNA. Peak read length is ~29 nt for all Ribo-Seq libraries, corresponding to the footprint of an elongating ribosome, whereas RNA-Seq libraries show a broad range of read lengths. (**D**) Schematic illustration showing how P sites were inferred from the metagene translatome. The distance from the 5′ end of reads of length 28 and 29 nt from the start and stop codon were identified and used as an offset for plotting P site position in **Figure 5**. Bars are coloured by the reading frame of the read 5′ end relative to the gene's start codon (frame 0; blue). Reads in green and red align out of frame.
